# Supplementary material for: Effect of active tuberculosis on the survival of HIV-infected adult patients who initiated antiretroviral therapy at public hospitals of Eastern Ethiopia: A retrospective cohort study
Source: PLoS One. 2022 Oct 31;17(10):e0277021. doi: 10.1371/journal.pone.0277021 (PMC9621427; doi:10.1371/journal.pone.0277021)
Supplement: S1 Table — (DOCX) [file pone.0277021.s001.docx]

**Questionnaire**

.NO. ---------------------------- Name of hospital ---------------------

Name and signature of data collector-------------------------------- Date -----------------------

**Section I:** Socio-Demographic characteristics.

| NO; | QUESTION | Coding and categories | REMARK |
| --- | --- | --- | --- |
| 101 | Age of the patient during ART initiation |  |  |
| 102 | Sex | 1. Male 2. Female |  |
| 103 | Religion | - - - 1. Muslim       2. Orthodox       3. Protestant       4. Others specify--------------- |  |
| 104 | Place of residence | 1) Urban 2) rural |  |
| 105 | Current Marital status: | 1. Married 2. Never married 2. Divorced 4. Widowed 3. Separated 9 Not recorded |  |
| 106 | Educational status: | 1. No formal education 2. Primary 3. Secondary 4. Tertiary 5. Other, specify-----------------   9 Not recorded |  |
| 107 | Occupation | --------------------------------------- |  |

**Section II;** Baseline Clinical and laboratory information

| NO; | QUESTION | Coding and categories | REMARK |
| --- | --- | --- | --- |
| 201 | Patient weight | --------------------------kg |  |
| 202 | Height | --------------------------cm |  |
| 203 | Body mass index | ----------------------kg/m^2^ |  |
| 204 | Patient WHO clinical stage while initiating ART | 1. Stage I 2. Stage II 3. Stage III 4. Stage IV   9.Not recorded |  |
| 205 | Was the patient TB o-infected at ART initiation? | 1. Yes 2. No | If 2 skip to 208 |
| 206 | Type of tuberculosis the patients developed | 1. Pulmonary tuberculosis 2. Extra pulmonary tuberculosis | If 2 or 9 skip to 208 |
| 207 | Pulmonary tuberculosis | 1. Smear positive 2. Smear negative |  |
| 208 | Patient functional status | 1. Working 2. Ambulatory 3. Bedridden   9 Not recorded |  |
| 209 | Hemoglobin level in g/dl | ----------------------------- |  |
| 210 | CD4 count level in cells/mm3 | ---------------------------- |  |
| 211 | Past opportunistic infections related to AIDS other than TB | 1. Zoster  2. Bacterial pneumonia  3. Thrush(oral/vaginal)  4. Diarrhea (chronic/acute)  5. Pneumocystis pneumonia  6. Ulcers (mouth/genital)  7. Cryptococcus meningitis  8. Other (specify)………………  9. No OPI |  |

**Section; III** Anti-TB, ART and Prophylactic medication

| NO; | Variable | Coding and categories | REMARK |
| --- | --- | --- | --- |
| 301 | Date ART started | _____/_____/__ dd/mm/yy E.C  ------/------/------------dd/mm/yy G.C |  |
| 302 | Was the patient co-infected with tuberculosis during follow up? | 1. Yes 2. No | If 2 skip to 306 |
| 303 | Date of TB diagnosis | ______/_____/__dd/mm/yy E.C  ------/------/------------dd/mm/yy G.C |  |
| 304 | Date of anti-TB initiation | ______/_____/__dd/mm/yy E.C  ------/------/------------dd/mm/yy G.C |  |
| 305 | Date of TB treatment completed | ______/_____/__dd/mm/yy E.C  ------/------/------------dd/mm/yy G.C |  |
| 306 | Initial ART regimen given | 1. 1c=AZT+3TC+NVP 2. 1d= AZT+3TC+EFV 3. 1e= TDF+3TC+EFV 4. 1f= TDF+3TC+NVP 5. 1g=ABC+3TC+EFV 6. 1h=ABC+3TC+NVP 7. Other(specify)-------- |  |
| 307 | Regimen change during the follow up time | - - - 1. Yes       2. No | **If 2 skip to q.no 309** |
| 308 | Reason for regimen change | --------------------------------------- |  |
| 309 | Adherence to ART drug | 1. Good 2. Fair 3. Poor   9 Not recoded |  |
| 310 | Did the patient prescribed  cotrimoxazole prophylactic therapy | 1. yes 2. No |  |

**Section IV** Patient’s follow up information to be filled from ART register and patient card.

| No. | VARIABLE | Coding and categories | REMARK |
| --- | --- | --- | --- |
| 401 | Patient last visit before December 31,2018 | _____/____/_______ dd/mm/yy E.C  ------/------/------------dd/mm/yy G.C |  |
| 402 | Patient status on December 31,2018 | 1. Dead 2. Transferred out 3. Loss to follow up 4. Alive / On treatment | If 2 or 3 or 4 or 5 skip to next section |
| 403 | Date of death | ____/______/____ dd/mm/yy E.C    ------/------/------------dd/mm/yy G.C |  |

**Section V:** Base line Social condition

| NO; | Variable | Coding and categories | REMARK |
| --- | --- | --- | --- |
| 501 | HIV serostatus  Disclosure | 1. Husband/ wife 2. Own child 3. Parents 4. Brother /sister 5. Relative 6. Nobody knew 7. Not recorded |  |
| 502 | Condition of husband/ wife | - - - 1. Health       2. Chronically ill       3. Dead       4. Unknown       5. Not recorded | If married |
| 503 | HIV status of husband or wife | 1. Positive  2. Negative  3. Unknown  9. Not recorded | If positive |
| 504 | Was he/she on ART? | 1.Yes  2. No |  |
