# Supplementary material for: Effect of active tuberculosis on the survival of HIV-infected adult patients who initiated antiretroviral therapy at public hospitals of Eastern Ethiopia: A retrospective cohort study
Source: PLoS One. 2022 Oct 31;17(10):e0277021. doi: 10.1371/journal.pone.0277021 (PMC9621427; doi:10.1371/journal.pone.0277021)
Supplement: Attachment — Submitted filename: Response to Reviewers.docx [file pone.0277021.s005.docx]

**Response to Reviewers**

**Manuscript ID:** PONE-D-22-21893R1

**Title:** **Effect of active tuberculosis on the survival of HIV-infected adult patients who initiated antiretroviral therapy at public hospitals of Eastern Ethiopia: A retrospective cohort study**

Respected Reviewers and Editors, We are really grateful for your quick response to our article. We appreciate your considerate and insightful comments and recommendations. Each comment and recommendation made by the editor and each reviewer has been addressed in the following sections with corrections and responses.

**Journal Requirements:**

Please review you reference list to ensure that it is complete and correct. If you have cited papers that have been retracted, please include the rationale for doing so in the manuscript text, or remove these references and replace them with relevant current references. Any changes to the reference list should be mentioned in the rebuttal letter that accompanies your revised manuscript. If you need to cite a retracted article, indicate the article’s status in the References list and also include a citation and full reference for the retraction notice.

**Author response:** Thank you so much for your important suggestion. We have checked the reference list for its completeness and correctness. We have not cited any retracted articles. We have cited reference number five (previously missed) in our discussion section of paragraph 4 (Page 17, line no. 276). Additionally, we have corrected reference 12. Thanks once again.

**Additional Editor Comments**:

Newly added Figure 1 explain the recruitment procedure, it is clear. Required sample size of N=566 was proportionally divided between 4 hospitals. Then number required from in each hospital was divided equally between exposed and unexposed groups. Then those 8 numbers were recruited randomly. Therefore it is incorrect to describe the sampling process as simple random sampling, or as random selection from each hospital. They have used a type of stratified sampling (random sampling stratified by hospital and exposure status).

**Author response:** Dear Editor, We are so grateful for your intellectual suggestions. Thanks a lot. What you mentioned is all about our sampling technique. We have corrected the previous description of the sampling technique as per your suggestion. As you stated, we used a random sampling stratified by hospital, and exposure status was used to select the study participants. First, the required sample was proportionally allocated to the four hospitals. Then the population in each hospital was stratified based on their exposure status. Finally, each study participant was selected randomly from each stratum. Thanks once again.

**Reviewer #2**

The authors have taken care of all observations and suggestions I made during my first review. The paper should be ready for publication, subject to the satisfaction of the other reviewers and the editor.

**Author response:** Dear Reviewer, Thanks for your time with our work.

**Reviewer #3**

The authors has adequately responded to the queries raised by the reviewer.

However, at the start of the discussion section some modification may be needed as commented below.

In the current study, the overall rate was 6.55 per 100 PYO in the entire follow-up period This finding is “comparable with a previous study conducted in Ethiopia”([13]; [11].

However, it is “higher compared to the studies conducted in Ethiopia” and other countries of African.

The part of these sentences…”comparable with a previous study conducted in Ethiopia”.. and …“higher compared to the studies conducted in Ethiopia” … are seems to be in disagreement and may need to be fixed.

**Author response:** Dear Reviewer, Thank you so much for your rational comment. We have corrected the mentioned section by specifying the particular study settings of those previous studies conducted in Ethiopia. It is corrected as “This finding is comparable with the previous studies conducted in the seven University Teaching Hospitals and Somali Regional State of Ethiopia[13]; [11].” “However, it is higher compared to the studies conducted in Jinka Hospital, South Omo zone of Ethiopia and other countries of Africa [14]; [20] : [21].”

**Reviewer #4**

Manuscript still has some grammatical errors that need to be corrected.

**Author response:** Dear Reviewer, Thanks for your suggestion. We have corrected all grammar errors throughout the manuscript.

Lines 241 to 251 offers conflicting information for instance.

**Author response:** Dear Reviewer, Thanks so much for you intellectual comments. We have corrected the mentioned section by specifying the particular study areas of those previous studies conducted in Ethiopia. It is corrected as “This finding is comparable with the previous studies conducted in the seven University Teaching Hospitals and Somali Regional State of Ethiopia[13]; [11].” “However, it is higher compared to the studies conducted in Jinka Hospital, South Omo zone of Ethiopia and other countries of Africa [14]; [20] : [21].”
